# Staged Surgery for Synchronous Proximal Gastric Adenocarcinoma and Pancreatic Head Ductal Adenocarcinoma with an Early Favorable Outcome: A Case Report

**DOI:** 10.3390/jcm15208119

**Published:** 2026-10-19

**Authors:** Pavel V. Sokolov, Mikhail B. Potievskiy, Danil V. Podluzhniy, Andrey B. Ryabov, Mikhail E. Rozhkov, Viltaliy Yu. Skoropad, Aleksei A. Nevolskikh, Sergei A. Ivanov, Peter V. Shegai, Andrei D. Kaprin

**Affiliations:** 1 FSBI “National Medical Research Radiological Center” of the Ministry of Health of the Russian Federation (FSBI “NMRRC”), 4 Koroleva Street, Obninsk 249036, Russia; sokolov-p-v@yandex.ru (P.V.S.); dr.podluzhny@mail.ru (D.V.P.); ryabovdoc@mail.ru (A.B.R.); mrrc@mrrc.obninsk.ru (M.E.R.); nevol@mrrc.obninsk.ru (A.A.N.); oncourolog@gmail.com (S.A.I.); kaprin@mail.ru (A.D.K.); 2 Department of Oncology and Roentgenoradiology named after Academician V. P. Kharchenko, Institute of Medicine, Peoples’ Friendship University of Russia—RUDN University, 6 Miklukho-Maklaya Street, Moscow 117198, Russia

**Keywords:** synchronous gastric and pancreatic cancer, proximal gastrectomy, double-tract reconstruction, pylorus-preserving pancreatoduodenectomy, neoadjuvant chemotherapy

## Abstract

**Background**. Synchronous primary gastric cancer and pancreatic ductal adenocarcinoma (PDAC) are uncommon, and published experience with their combined treatment is limited. Proximal gastric adenocarcinoma combined with pancreatic head PDAC presents a particular challenge because resection and reconstruction must address two anatomically separate primary tumors. **Materials and Methods**. A 62-year-old man with poorly differentiated proximal gastric adenocarcinoma (cT3N0M0) and pancreatic head PDAC (cT3N1M0) received six cycles of neoadjuvant FOLFIRINOX. Preoperative ECOG performance status was 0. Treatment comprised proximal gastrectomy with D2 lymphadenectomy and double-tract reconstruction and, approximately 10 weeks later, pylorus-preserving pancreatoduodenectomy. Contrast-enhanced CT before the second stage showed no disease progression. **Results**. Intra-abdominal hemorrhage after proximal gastrectomy required relaparotomy. Histopathological examination confirmed microscopically negative resection margins (R0) for both tumors. Gastric tumor regression was Becker grade 3, indicating minimal or no regression; pancreatic regression was Modified Ryan score 3, indicating a poor or absent response. Six cycles of adjuvant modified FOLFIRINOX were reported. At the last reported follow-up, 10 months after the second operation, the patient had no evidence of progression and an ECOG performance status of 0. **Conclusions**. This case documents the technical feasibility of staged gastric and pancreatic resection after neoadjuvant chemotherapy in a patient with preoperative ECOG performance status 0, with histologically negative resection margins and an early favorable outcome despite a postoperative complication. The reported R0 status describes the morphological assessment of resection margins. Longer follow-up is required to assess durable disease control.

## 1. Introduction

Gastric cancer and pancreatic ductal adenocarcinoma (PDAC) remain important causes of cancer-related mortality worldwide. GLOBOCAN 2022 estimated approximately 968,000 new gastric cancers and 660,000 gastric cancer deaths, and approximately 511,000 new pancreatic cancers and 467,000 pancreatic cancer deaths [1]. These burdens underscore the need for careful multidisciplinary management when the two malignancies coexist.

Synchronous primary gastric cancer and PDAC are uncommon. Published reports include heterogeneous gastric locations, pancreatic sites, histological types, and treatment strategies [2,3,4,5,6,7,8,9,10,11,12,13,14,15,16]. This literature should not be treated as a uniform cohort of invasive PDAC, because some reports concern acinar cell carcinoma, sarcomatoid carcinoma, pancreatic carcinoma in situ, or additional metastatic lesions.

Therefore there are no evidence-based recommendations regarding the optimal treatment workflow, sequencing of drug treatment and surgery, the extent of gastric resection, reconstruction strategy, or the feasibility of simultaneous versus staged surgical treatment. Consequently, therapeutic decision-making relies largely on multidisciplinary discussion and personalized treatment strategy [17,18].

Management requires balancing the extent of oncological resection against postoperative gastrointestinal function and recovery. Simultaneous gastric and pancreatic resections are feasible in selected patients, but their acceptability depends on the resections required, nutritional status, comorbidity, and reconstruction. Evidence for proximal gastrectomy with double-tract reconstruction is strongest in selected gastric cancer populations and cannot by itself establish the adequacy of this approach for synchronous locally advanced gastric cancer and PDAC [19,20,21,22,23].

Here, we report neoadjuvant FOLFIRINOX followed by staged proximal gastrectomy with double-tract reconstruction and pylorus-preserving pancreatoduodenectomy for synchronous proximal gastric adenocarcinoma and pancreatic head PDAC. The report focuses on treatment sequencing, reconstruction, and the limits of an early outcome.

## 2. Case Presentation

### 2.1. Diagnosis and Staging

A 62-year-old male patient was referred to A.F. Tsyb Medical Radiological Research Center in December 2024 because of progressive epigastric pain and an unintentional 15-kg weight loss over the preceding two months. His medical history was significant for stage I laryngeal squamous cell carcinoma (pT1aN0M0), successfully treated by partial laryngectomy in 2021, with no evidence of recurrence.

**Figure 1:**
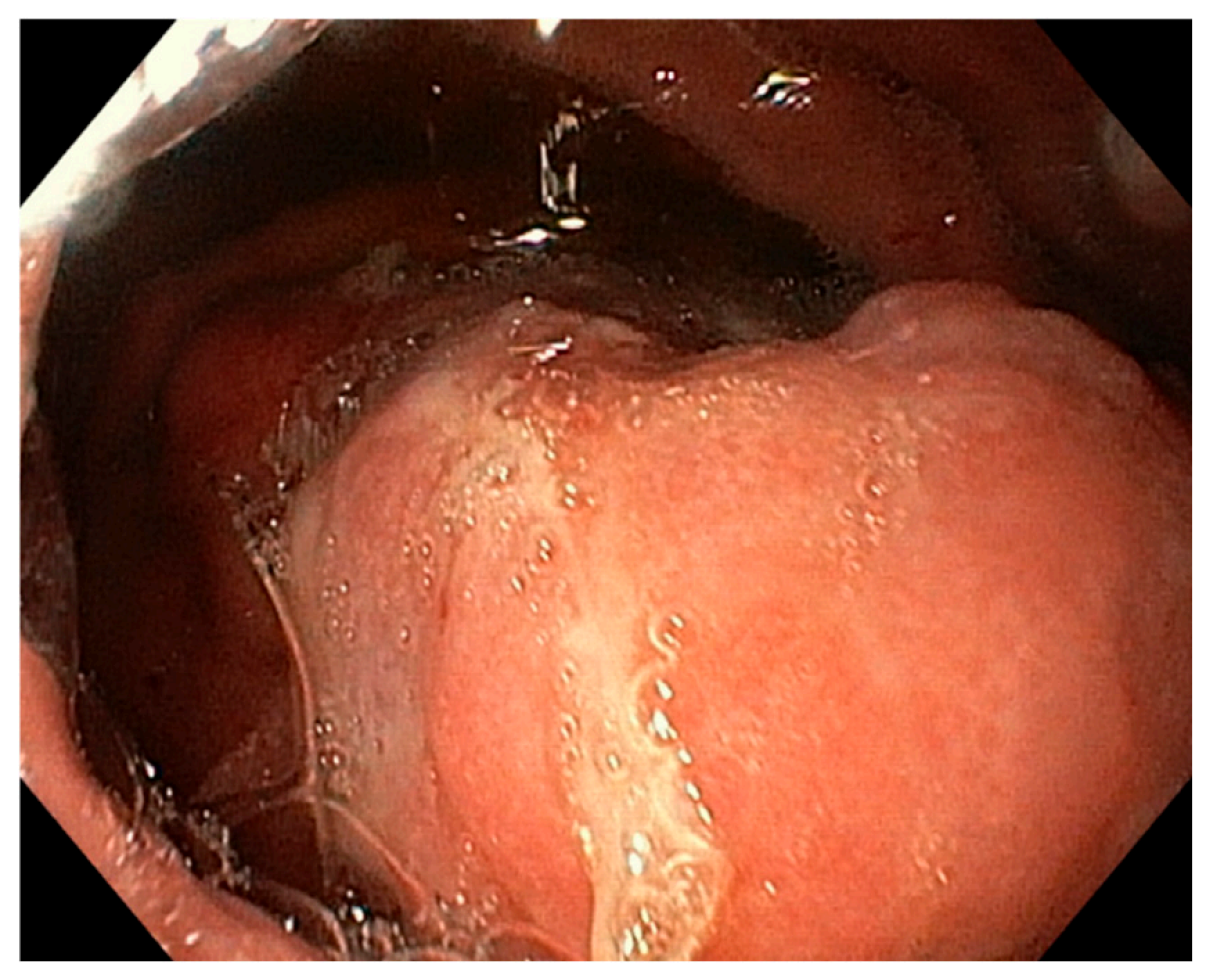
Endoscopic visualization of gastric tumor.

Upper gastrointestinal endoscopy revealed a broad-based exophytic lesion measuring approximately 40 × 50 mm located on the posterior wall of the proximal stomach, involving the upper third of the gastric body and extending toward the subcardial region. The superior margin of the tumor was approximately 2.5 cm distal to the esophagogastric junction (Figure 1). Histopathological examination of the endoscopic biopsy demonstrated poorly differentiated gastric adenocarcinoma.

**Figure 2:**
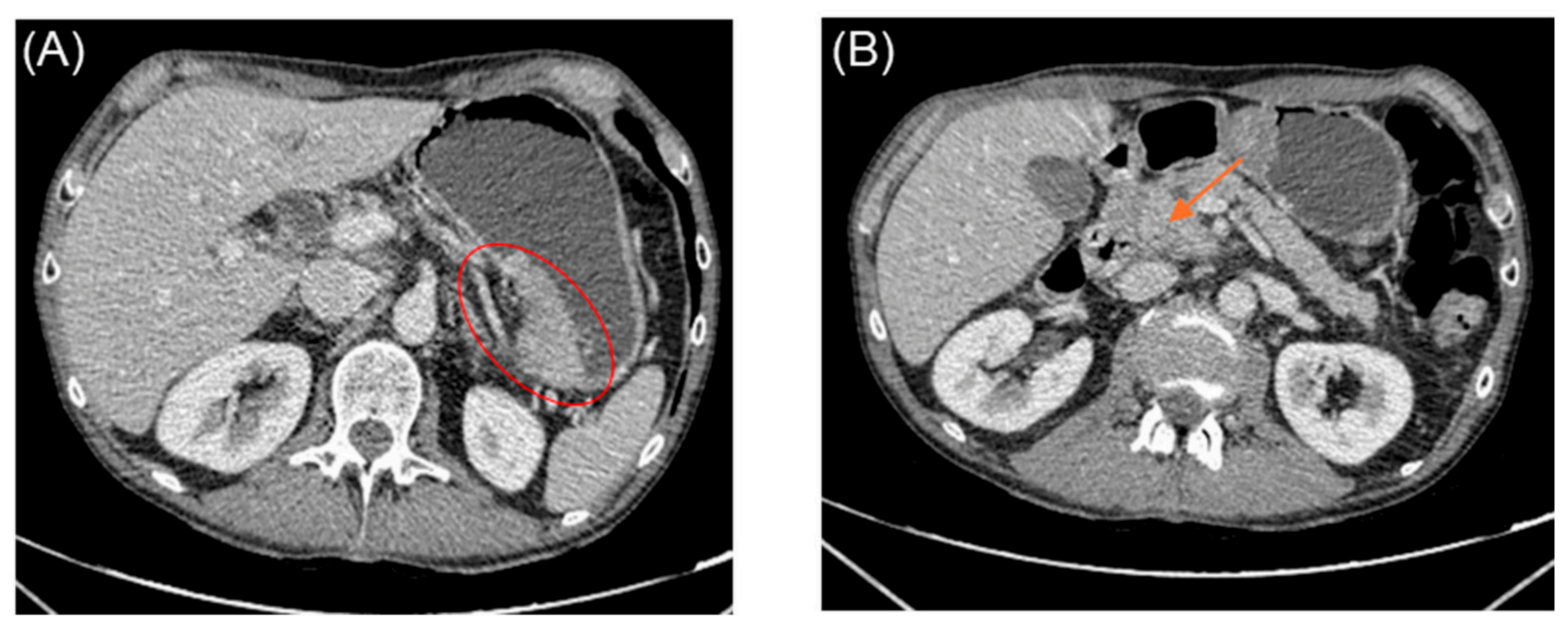
CT visualization of the tumors: (**A**)—gastric tumor, marked with a red circle; (**B**)—pancreatic head tumor, marked in orange.

Contrast-enhanced CT demonstrated the proximal gastric tumor and a 42 × 31 mm pancreatic head mass with enlarged peripancreatic lymph nodes (Figure 2A,B). Whole-body 18F-FDG PET/CT showed a gastric SUVmax of 5.0 and a pancreatic SUVmax of 1.6. Contrast-enhanced CT showed no involvement of the superior or inferior mesenteric arteries and veins, the celiac trunk, the common or proper hepatic artery, or the portal vein by the pancreatic tumor.

Endoscopic ultrasound (EUS) was performed with EUS-guided core needle biopsy of the pancreatic lesion, which confirmed PDAC. Thus, pancreatic tissue diagnosis was obtained before systemic treatment. Diagnostic laparoscopy found no macroscopic peritoneal carcinomatosis or distant metastatic disease. Peritoneal washing cytology is not documented in the available case information.

Serum tumor markers demonstrated only a mild elevation of carcinoembryonic antigen (CEA, 5.91 ng/mL; reference range < 4.70 ng/mL), whereas carbohydrate antigen 19-9 (CA19-9, 19.54 U/mL) and carbohydrate antigen 72-4 (CA72-4, 0.69 U/mL) were within normal limits.

Thus, based on the clinical, radiological, and histopathological data, the patient was diagnosed with three primary malignancies:

Poorly differentiated adenocarcinoma of the proximal stomach (cT3N0M0);

Pancreatic ductal adenocarcinoma of the pancreatic head (cT3N1M0);

History of stage I laryngeal squamous cell carcinoma (pT1aN0M0), resected in 2021 without evidence of recurrence.

### 2.2. Treatment

The case was discussed at a multidisciplinary tumor board. Six cycles of neoadjuvant FOLFIRINOX were selected to prioritize systemic treatment of PDAC while treating both gastrointestinal adenocarcinomas. Evidence for FOLFIRINOX in PDAC informed this decision [24]; its use here should not be interpreted as an established perioperative regimen for gastric cancer, for which FLOT-based evidence is available [25,26]. Treatment was reported to be well tolerated without clinically significant toxicity. Restaging using RECIST version 1.1 [27] showed stable disease in both tumors and no distant metastases.

After neoadjuvant chemotherapy, the multidisciplinary team considered simultaneous and staged resections. Preoperative ECOG performance status was 0, indicating preserved functional status. The staged strategy was selected on anatomical and technical grounds: the combination of a separate proximal gastric primary and a pancreatic head primary required coordinated resection, preservation of distal gastric perfusion, and reconstruction. ECOG 0 supported consideration of operative treatment but did not determine whether the two resections should be performed simultaneously or sequentially. Conventional pancreatoduodenectomy with distal gastric resection does not reproduce the anatomical requirements of a separate proximal gastric primary.

On 2 June 2025, proximal gastrectomy with D2 lymphadenectomy and double-tract reconstruction was performed without splenectomy. Review of the operative record confirmed dissection of lymph node stations Nos. 1, 2, 3a, 4sa, 4sb, 7, 8a, 9, 11p, and 11d. Lymphatic tissue was retrieved from all of these D2 stations and submitted for pathological examination. Intra-abdominal hemorrhage required emergency relaparotomy and ligation of a gastric vein. The patient subsequently recovered and was discharged on postoperative day 10 in satisfactory condition.

Histopathological examination demonstrated poorly differentiated gastric adenocarcinoma with Becker grade 3 regression, indicating minimal or no regression and more than 50% residual viable tumor within the tumor bed [28]. Residual carcinoma invaded the muscularis propria and showed lymphovascular and perineural invasion. All 13 examined regional lymph nodes were negative (9 from the lesser omentum and 4 from the greater omentum). The relatively low yield of 13 identifiable nodes may reflect treatment-related lymph node involution following neoadjuvant chemotherapy, despite tissue retrieval from all documented D2 stations [29]. Histopathological examination confirmed microscopically negative resection margins (R0), and the reported pathological classification was ypT2N0. The R0 designation in this report describes the morphological assessment of resection margins; the nodal findings are reported separately.

**Figure 3:**
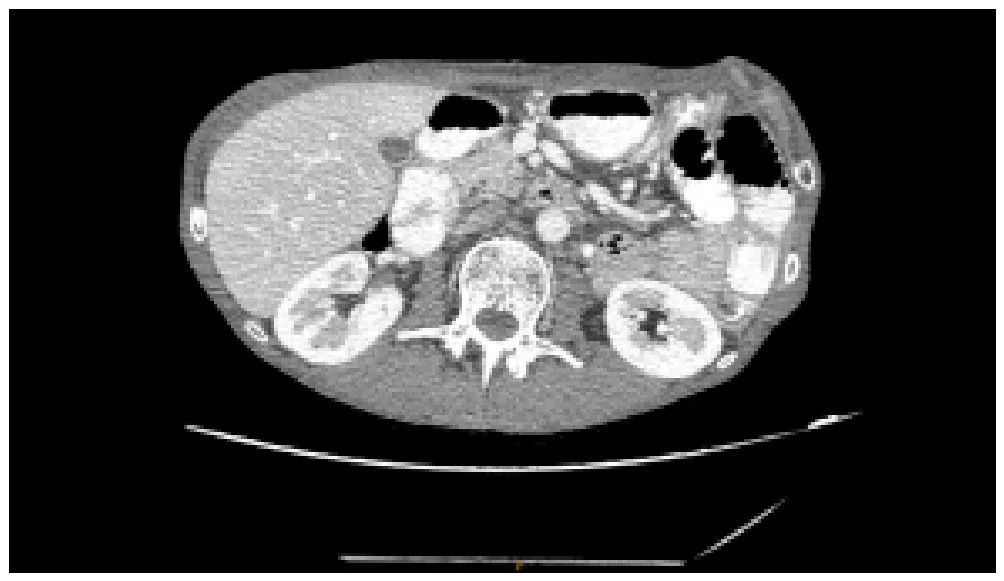
CT visualization after gastric resection.

Before the second stage, contrast-enhanced CT of the chest, abdomen, and pelvis was performed on 6 August 2025 and showed no evidence of disease progression (Figure 3). The CT preceded the second operation by six days. These findings supported proceeding with the planned second-stage resection.

On 12 August 2025, approximately 10 weeks after proximal gastrectomy, pylorus-preserving pancreatoduodenectomy was performed. Exploration showed a tumor involving the uncinate process and enlarged, firm peripancreatic nodes. The pancreatic parenchyma was soft. Preservation of the right gastric and right gastroepiploic arterial supply was described as a key technical objective for perfusion of the retained distal stomach.

**Figure 4:**
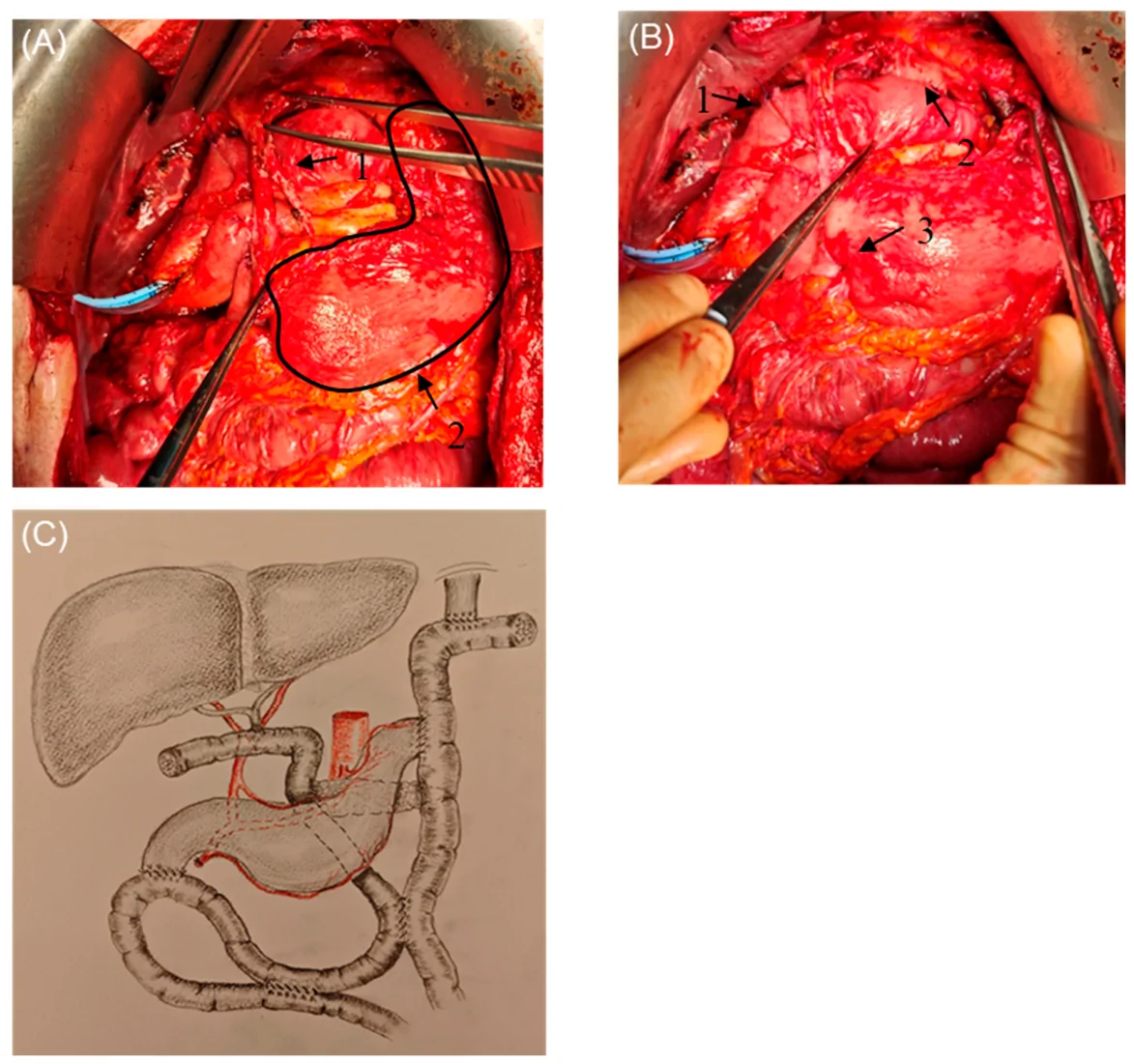
Pancreatoduodenal resection. (**A**)—Intraoperative view before reconstruction. (**B**)—Intraoperative view after reconstruction: 1—Hepaticojejunoanastomosis; 2—Pancreaticojejunoanastomosis; 3—Pylorojejunoanastomosis. (**C**)—Schema of reconstruction stage.

Pancreaticojejunostomy and hepaticojejunostomy were constructed using a jejunal loop originating from the ligament of Treitz. Adhesions and deformation after the preceding gastric operation limited the length available for pylorojejunostomy, which was therefore constructed using an additional jejunal limb (Figure 4).

Histopathological examination confirmed moderately differentiated PDAC (G2), with metastases in 2 of 16 regional lymph nodes. Histopathological examination confirmed microscopically negative resection margins (R0). The reported post-treatment classification was ypT2N1, with no clinical evidence of distant metastasis. Modified Ryan tumor regression score 3 indicated extensive viable tumor with a poor or absent treatment response [30].

Adjuvant modified FOLFIRINOX was started on 11 October 2025; six cycles were reported. At the last reported follow-up, 10 months after the second operation, the patient had no evidence of disease progression, had returned to normal daily activities, and had an ECOG performance status of 0. Nutritional status was described clinically as adequate, although serial weight, laboratory nutritional measures, and validated quality-of-life assessments were not provided.

## 3. Discussion

Reports of synchronous gastric and pancreatic malignancies are heterogeneous [2,3,4,5,6,7,8,9,10,11,12,13,14,15,16]. Table 1 presents selected published cases with outcome information verified from the primary reports, together with the present case. It is a descriptive comparison rather than an exhaustive systematic review or survival analysis. Differences in histology, disease extent, treatment, and follow-up preclude conclusions about the superiority of a particular surgical strategy.

Pancreatic body and tail lesions may be addressed with distal pancreatectomy, whereas a pancreatic head lesion may require pancreatoduodenectomy. Published experience demonstrates that simultaneous resections are possible in selected patients. Accordingly, the present staged approach should be evaluated as an individualized technical option rather than as a generally safer alternative.

The combination of a proximal gastric primary and pancreatic head PDAC creates specific problems of gastric perfusion and reconstruction. The present report adds experience with a staged double-tract and pylorus-preserving reconstruction.

### 3.1. Surgical Strategy and Oncological Adequacy

Proximal gastrectomy with double-tract reconstruction may offer functional benefits in selected patients [19,20,21,22,23]. In the setting of two synchronous primaries, the extent of gastric resection, the anatomical distribution of lymphadenectomy, preservation of gastric perfusion, and microscopic margin status should be considered as distinct components of treatment planning.

Gastric lymphadenectomy is defined by the anatomical stations dissected, rather than by the number of nodes subsequently identified in the specimen. The Japanese guidelines recommend D2 for cT2 or higher tumors; for proximal gastrectomy, stations 11p and 11d along the splenic artery form part of that anatomical definition [22,31]. The operative record documented dissection of Nos. 1, 2, 3a, 4sa, 4sb, 7, 8a, 9, 11p, and 11d, corresponding to the station-based definition of D2 for proximal gastrectomy [22,31]. Lymphatic tissue was retrieved from all of these stations and submitted for pathological examination. Neoadjuvant therapy can reduce the number of identifiable lymph nodes through treatment-related involution, stromal atrophy, fibrosis, and nodal shrinkage [29]. These changes provide a plausible explanation for the yield of 13 nodes in this patient despite retrieval from all documented D2 stations; however, the causal contribution of involution was not established by the available histopathological report. The 13 nodes reported by histopathology represent the nodes identified in the specimen and do not redefine the documented anatomical extent of dissection as D1. Randomized evidence also requires attention to the population studied: the Italian D1-versus-D2 trial found no overall-population survival difference, but long-term disease-specific outcomes favored D2 in patients with advanced, node-positive disease [32]. These findings support stage-appropriate lymphadenectomy rather than an assertion that D1 and D2 are interchangeable. In the present case, D2 lymphadenectomy was documented in the operative record, and histopathology demonstrated 13 negative regional nodes and microscopically negative resection margins. We use R0 specifically to report the histological margin result, rather than to make a comparative claim about lymphadenectomy or long-term disease control.

Splenic preservation should be distinguished from the extent of lymph node dissection. Stations 11p and 11d lie along the splenic artery, whereas station 10 is located at the splenic hilum; removal of these nodal tissues does not inherently require splenectomy [22,31]. In JCOG0110, 505 patients undergoing total gastrectomy for proximal gastric cancer without greater-curvature invasion were randomized to splenectomy or spleen preservation. Five-year overall survival was 75.1% and 76.4%, respectively; spleen preservation was noninferior, while splenectomy increased morbidity [33]. Current Japanese guidelines therefore support spleen preservation in this setting, rather than mandatory prophylactic splenectomy for proximal location alone [31]. An alternative view concerns the value of additional hilar-node clearance: the five-year post hoc analysis of Fuges-02 reported better disease-free survival with D2 plus station 10 dissection than with D2 alone (63.9% versus 55.1%) in advanced proximal tumors without greater-curvature invasion [34]. Importantly, that dissection was spleen-preserving. Thus, the debate concerns which nodal stations should be cleared, not whether splenectomy is universally necessary. Both trials involved total gastrectomy and should not be taken as direct validation of every aspect of the present proximal gastrectomy. Nevertheless, they provide evidence against the proposition that spleen preservation alone makes a proximal gastric resection noncurative. The described posterior-wall/subcardial location is not, in itself, an indication for mandatory splenectomy.

The intended functional benefit of retaining the distal stomach was relevant to the subsequent pancreatoduodenectomy and adjuvant therapy. However, neither nutritional benefit nor lower morbidity can be established from this observation. The patient required reoperation for hemorrhage, and adhesions complicated reconstruction at the second stage.

A staged approach also leaves one known primary tumor in situ during recovery and entails repeat abdominal surgery. Interval progression, peritoneal dissemination, adhesions, and cumulative morbidity are relevant concerns. Baseline laparoscopy was negative for macroscopic peritoneal disease, and CT six days before the second operation showed no progression. These observations do not exclude microscopic dissemination or demonstrate that staging does not increase oncological risk. The approximately 10-week interval requires consideration when balancing recovery against treatment delay.

FOLFIRINOX was chosen as an individualized compromise that prioritized PDAC treatment [24]. Reference [24] reports metastatic PDAC and does not directly validate perioperative treatment of two synchronous primaries. Gastric perioperative treatment has a separate evidence base [25,26]. Stable disease during treatment permitted subsequent surgery in this patient, but does not establish comparative regimen efficacy.

Both tumors showed limited histological regression: Becker grade 3 in the stomach and Modified Ryan score 3 in the pancreas [28,30]. The systems use different criteria and should not be directly equated numerically, but neither finding supports a major pathological response. No substantial difference in chemotherapy sensitivity can be inferred from these findings.

Histopathological examination confirmed microscopically negative resection margins (R0) for both tumors. This is the specific pathological endpoint reported here. Absence of progression at 10 months constitutes an early favorable observation; longer follow-up is needed to assess recurrence and survival, particularly given residual viable tumor and pancreatic nodal involvement.

### 3.2. Clinical Implications

Synchronous gastric cancer and pancreatic ductal adenocarcinoma are exceedingly uncommon; therefore, prospective studies are unlikely to become available. Carefully documented clinical observations remain valuable sources of evidence for surgical decision-making.

This case illustrates the feasibility of completing staged gastric and pancreatic resections and subsequent systemic treatment in one selected patient. Treatment planning must consider preoperative functional and nutritional status, vessel involvement, nodal clearance, preservation of gastric perfusion, and the consequences of the interval between operations. The report cannot define selection criteria or recommend this approach over simultaneous resection.

### 3.3. Limitations

This is a single case without a comparator and with only 10 months of reported follow-up after the second operation. Preoperative ECOG performance status was 0, but quantitative nutritional outcomes were not available in the supplied record. The report describes histological margin status and regional nodal findings; it cannot compare alternative lymphadenectomy strategies or establish long-term disease control. Both regression scores indicate limited treatment response. CT without progression cannot exclude microscopic peritoneal disease. The literature comparison is selective and includes biologically different pancreatic neoplasms, so survival outcomes cannot be pooled or used to infer comparative effectiveness.

### 3.4. Patient Perspective

From the treating team’s perspective, the patient achieved a favorable early clinical and functional outcome after completion of the staged surgical treatment and adjuvant chemotherapy. At the last reported follow-up, 10 months after the second operation, there was no evidence of disease progression, and ECOG performance status was 0, consistent with full activity without restriction in usual daily activities. These findings indicate recovery of functional status despite the postoperative hemorrhage requiring relaparotomy after the first operation. Continued surveillance is required to assess the durability of disease control. This assessment reflects the treating physicians’ clinical evaluation.

## 4. Conclusions

We report the first documented case of synchronous proximal gastric adenocarcinoma and pancreatic head ductal adenocarcinoma treated with neoadjuvant FOLFIRINOX followed by staged function-preserving surgery. Individualized multidisciplinary planning enabled R0 resection while preserving gastrointestinal function and allowing timely administration of adjuvant chemotherapy. No progression was reported 10 months after the second operation. Although conclusions cannot be drawn from a single observation, this case suggests that staged organ-preserving surgery may represent a feasible treatment option for carefully selected patients with this exceptionally rare combination of malignancies.

## Figures and Tables

**Table 1:** Selected reports of synchronous gastric and pancreatic neoplasms with reported outcomes.

Report	Pancreatic Histology and Site	Treatment	Reported Follow-Up and Outcome
Fang 2021 [11]	Acinar cell carcinoma; tail	Distal gastric and pancreatic resection; SOX	No recurrence or metastasis as of March 2021; a duration is not supplied in the outcome statement.
Inoue 2021 [12]	Carcinoma in situ/PanIN-3; body/tail	Total gastrectomy, distal pancreatectomy and splenectomy	No recurrence at 2 years; postoperative malnutrition prevented adjuvant chemotherapy.
Shupp 2022 [13]	IPMN-associated adenocarcinoma; head	Extended Whipple; attempted chemoradiation	Clinical deterioration and death after treatment intolerance; exact survival interval not reported. Residual gastric disease was described.
Ebihara 2024 [15]	Invasive ductal carcinoma; tail	Total gastrectomy and distal pancreatosplenectomy	No recurrence on reported postoperative CT/PET-CT; follow-up duration not specified.
Lu 2025 [16]	Sarcomatoid carcinoma; tail	Gastric ESD and pancreatic resection	Liver metastases and death on postoperative day 107.
Present case	PDAC; head	Neoadjuvant FOLFIRINOX; staged resections; adjuvant mFOLFIRINOX	No progression at 10 months after second operation; ECOG 0.

PDAC, pancreatic ductal adenocarcinoma; SOX, S-1 plus oxaliplatin; IPMN, intraductal papillary mucinous neoplasm; PanIN-3, high-grade pancreatic intraepithelial neoplasia; ESD, endoscopic submucosal dissection. Reported observations are not estimates of median or long-term survival. Cases with non-PDAC histology are identified explicitly.

## Data Availability

The original contributions presented in this study are included in the article. Further inquiries can be directed to the corresponding author.

## References

[1] Bray F., Laversanne M., Sung H., Ferlay J., Siegel R.L., Soerjomataram I., Jemal A. (2024). Global cancer statistics 2022: GLOBOCAN estimates of incidence and mortality worldwide for 36 cancers in 185 countries. CA Cancer J. Clin..

[2] Eriguchi N., Aoyagi S., Hara M., Okuda K., Tamae T., Fukuda S., Hashino K., Hashimoto M., Sato S., Furukawa S. (2000). A case of synchronous double cancers of the pancreas and stomach. Kurume Med. J..

[3] Kubota E., Kataoka H., Hayashi K., Kamiya T., Sasaki M., Ogasawara N., Yamada T., Wada T., Mori Y., Mizoshita T. (2009). Advanced stomach and pancreas cancer successfully treated with combination chemotherapy with S-1/paclitaxel/lentinan. Hepatogastroenterology.

[4] Muroni M., D’Angelo F., Pezzatini M., Sebastiani S., Noto S., Pilozzi E., Ramacciato G. (2010). Synchronous gastric adenocarcinoma and pancreatic ductal adenocarcinoma. Hepatobiliary Pancreat. Dis. Int..

[5] Kourie H.R., Markoutsaki N., Roussel H., Rahmi G., van der Stiegel M., Palazzo L., Fabre M., Cuenod C.A., Dubreuil O., Landi B. (2013). Double pancreatic and gastric adenocarcinomas: A rare association. Clin. Res. Hepatol. Gastroenterol..

[6] Ohtsubo K., Ishikawa D., Nanjo S., Takeuchi S., Yamada T., Mouri H., Yamashita K., Yasumoto K., Gabata T., Matsui O. (2013). Synchronous triple cancers of the pancreas, stomach, and cecum treated with S-1 followed by pancrelipase treatment of pancreatic exocrine insufficiency. JOP J. Pancreas.

[7] Santos-Fernández J., Arenal-Vera J.J., Cítores-Pascual M.Á., Fernández-Orcajo P., De-Benito-Sanz M., Benito-Fernández C., Tinoco-Carrasco C. (2015). Synchronic gastric and pancreatic ductal adenocarcinoma. A case report. Rev. Esp. Enferm. Dig..

[8] Ghothim M., Havlík R., Skalický P., Klos D., Vrba R., Strážnická J., Skopal L., Neoral Č., Loveček M. (2015). Synchronous cancer duplicities of pancreas and stomach/kidney and their surgical treatment. Rozhl. Chir..

[9] Yokoyama Y., Sakata H., Uekusa T., Tajima Y., Ishimaru M. (2020). Solitary pancreatic metastasis of gastric cancer with synchronous pancreatic ductal carcinoma: A case report. Int. J. Surg. Case Rep..

[10] Ma Y.H., Yamaguchi T., Yasumura T., Kuno T., Kobayashi S., Yoshida T., Ishida T., Ishida Y., Takaoka S., Fan J.L. (2021). Pancreatic cancer secondary to intraductal papillary mucinous neoplasm with collision between gastric cancer and B-cell lymphoma: A case report. World J. Clin. Cases.

[11] Fang T., Liang T.T., Wang Y.Z., Wu H.T., Liu S.H., Wang C. (2021). Synchronous concomitant pancreatic acinar cell carcinoma and gastric adenocarcinoma: A case report and review of the literature. World J. Clin. Cases.

[12] Inoue M., Hakoda K., Sawada H., Hotta R., Ohmori I., Miyamoto K., Toyota K., Sadamoto S., Takahashi T. (2021). Synchronous gastric cancer and carcinoma in situ of the pancreas. Clin. Case Rep..

[13] Shupp B., Liaquat H., Rollins S., Stoll L., Singh G., Quiros R.M., Matin A. (2022). A rare case of synchronous intraductal papillary mucinous neoplasm-associated pancreatic adenocarcinoma and signet ring cell gastric adenocarcinoma. Am. J. Case Rep..

[14] Read C., Hughes M., Itani O. (2023). Presentation of gastrointestinal stromal tumor, pancreatic adenocarcinoma, and gastric adenocarcinoma in a woman with no identifiable genetic abnormalities. Am. Surg..

[15] Ebihara M., Fujisawa K., Haruta S., Uruga H., Ueno M. (2024). Laparoscopic radical total gastrectomy and pancreatosplenectomy for synchronous cancer of the stomach and pancreas. Cureus.

[16] Lu G., Tu J., Jiang R. (2025). Case report: Synchronous pancreatic sarcomatoid carcinoma and gastric cancer: A case study with literature review. Front. Immunol..

[17] National Comprehensive Cancer Network (2025). NCCN Clinical Practice Guidelines in Oncology: Pancreatic Adenocarcinoma.

[18] Kudashkin N.E., Gladkov O.A., Zagaynov V.E., Kuchin D.M., Lyadov V.K., Podluzhny D.V., Pokataev I.A., Tryakin A.A., Fedyanin M.Y., Chernykh M.V. (2024). Pancreatic cancer. Malig. Tumours.

[19] Zhu G., Jiao X., Zhou S., Zhu Q., Yu L., Sun Q., Li B., Fu H., Huang J., Lang W. (2024). Can proximal gastrectomy with double-tract reconstruction replace total gastrectomy? A meta-analysis of randomized controlled trials and propensity score-matched studies. BMC Gastroenterol..

[20] Li B., Wang Y., Li B., Shan F., Li Z. (2024). Short-term outcomes and long-term quality of life of reconstruction methods after proximal gastrectomy: A systematic review and meta-analysis. BMC Cancer.

[21] Zhang Y., Zhang H., Yan Y., Ji K., Jia Z., Yang H., Fan B., Wang A., Wu X., Zhang J. (2023). Double-tract reconstruction is superior to esophagogastrostomy in controlling reflux esophagitis and enhancing quality of life after proximal gastrectomy: Results from a prospective randomized controlled clinical trial in China. Chin. J. Cancer Res..

[22] Japanese Gastric Cancer Association (2023). Japanese gastric cancer treatment guidelines 2021 (6th edition). Gastric Cancer.

[23] Hirata Y., Kim H.I., Grotz T.E., Matsuda S., Badgwell B.D., Ikoma N. (2022). The role of proximal gastrectomy in gastric cancer. Chin. Clin. Oncol..

[24] Conroy T., Desseigne F., Ychou M., Bouché O., Guimbaud R., Bécouarn Y., Adenis A., Raoul J.L., Gourgou-Bourgade S., De La Fouchardière C. (2011). FOLFIRINOX versus gemcitabine for metastatic pancreatic cancer. N. Engl. J. Med..

[25] Al-Batran S.E., Homann N., Pauligk C., Goetze T.O., Meiler J., Kasper S., Kopp H.G., Mayer F., Haag G.M., Luley K. (2019). Perioperative chemotherapy with fluorouracil plus leucovorin, oxaliplatin, and docetaxel versus fluorouracil or capecitabine plus cisplatin and epirubicin for locally advanced, resectable gastric or gastro-oesophageal junction adenocarcinoma (FLOT4): A randomised, phase 2/3 trial. Lancet.

[26] Lordick F., Carneiro F., Cascinu S., Fleitas T., Haustermans K., Piessen G., Vogel A., Smyth E.C., ESMO Guidelines Committee (2022). Gastric cancer: ESMO Clinical Practice Guideline for diagnosis, treatment and follow-up. Ann. Oncol..

[27] Eisenhauer E.A., Therasse P., Bogaerts J., Schwartz L.H., Sargent D., Ford R., Dancey J., Arbuck S., Gwyther S., Mooney M. (2009). New response evaluation criteria in solid tumours: Revised RECIST guideline (version 1.1). Eur. J. Cancer.

[28] Becker K., Mueller J.D., Schulmacher C., Ott K., Fink U., Busch R., Böttcher K., Siewert J.R., Höfler H. (2003). Histomorphology and grading of regression in gastric carcinoma treated with neoadjuvant chemotherapy. Cancer.

[29] Lai H., Zheng J., Li Y. (2022). Comparison of Four Lymph Node Staging Systems in Gastric Adenocarcinoma after Neoadjuvant Therapy—A Population-Based Study. Front. Surg..

[30] (2024). Protocol for the Examination of Specimens from Patients with Carcinoma of the Exocrine Pancreas. College of American Pathologists Cancer Protocol. Version 5.0.0.0. https://www.cap.org/wp-content/uploads/protocols/Panc.Endo_5.0.1.0.REL_CAPCP.pdf?download=true.

[31] Japanese Gastric Cancer Association (2026). Japanese gastric cancer treatment guidelines 2025 (7th edition). Gastric Cancer.

[32] Degiuli M., Reddavid R., Tomatis M., Ponti A., Morino M., Sasako M., Rebecchi F., Garino M., Vigano L., Scaglione D. (2021). D2 dissection improves disease-specific survival in advanced gastric cancer patients: 15-year follow-up results of the Italian Gastric Cancer Study Group D1 versus D2 randomised controlled trial. Eur. J. Cancer.

[33] Sano T., Sasako M., Mizusawa J., Yamamoto S., Katai H., Yoshikawa T., Nashimoto A., Ito S., Kaji M., Imamura H. (2017). Randomized Controlled Trial to Evaluate Splenectomy in Total Gastrectomy for Proximal Gastric Carcinoma. Ann. Surg..

[34] Lin J., Xu B., Zheng H., Li P., Xie J.W., Wang J.B., Lu J., Chen Q.Y., Cao L.L., Lin M. (2024). Laparoscopic Spleen-Preserving Hilar Lymphadenectomy for Advanced Proximal Gastric Cancer Without Greater Curvature Invasion: Five-Year Outcomes from the Fuges-02 Randomized Clinical Trial. JAMA Surg..

